# Comparing engineered nuclear-localized reporter cassettes

**DOI:** 10.17912/micropub.biology.001014

**Published:** 2023-11-02

**Authors:** HaoSheng Sun, Isabel Beets, William Schafer, Oliver Hobert

**Affiliations:** 1 Cell, Developmental and Integrative Biology, University of Alabama at Birmingham, Birmingham, Alabama, United States; 2 Howard Hughes Medical Institute, Chevy Chase, Maryland, United States; 3 Division of Animal Physiology and Neurobiology, KU Leuven, Leuven, Flanders, Belgium; 4 Neurobiology division, MRC Laboratory of Molecular Biology, Cambridge, England, United Kingdom; 5 Biological Sciences, Columbia University, New York, New York, United States

## Abstract

Recent single-cell transcriptome analysis has revealed a tremendous breadth and specificity of neuropeptide-encoding gene expression in the nervous system of *C. elegans.* To analyze the dynamics of neuropeptide gene expression, as well as to dissect the regulatory mechanism by which their expression is controlled, reporter genes remain an important tool. Using CRISPR/Cas9 genome-engineering, we generate here reporter alleles for 6 different neuropeptide encoding genes (3 *flp* genes, 1 *nlp* and 2 insulin genes). We find that different reporter cassettes result in different levels of reporter expression and recommend usage of an SL2::GFP::H2B or GFP::H2B::SL2 cassette.

**Figure 1. Comparing engineered nuclear-localized reporter cassettes:**
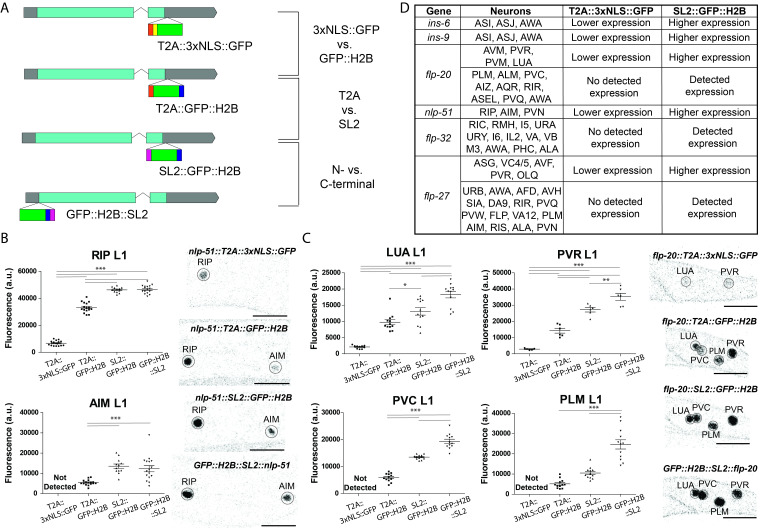
Fig 1. (A) Schematic of reporter cassette comparison. (B) Comparison of the different reporter cassettes for neuropeptide *nlp-51* . Analysis for *nlp-51* GFP fluorescence in the RIP and AIM neurons is shown on the left, with representative images on the right. (C) Comparison of the different reporter cassettes for neuropeptide *flp-20* . Analysis for *flp-20* GFP fluorescence in the LUA, PVR, PVC, and PLM neurons is shown on the left, with representative images on the right. For B and C, *P<0.05, **P<0.01, ***P<0.001 posthoc t-test with Tukey multiple comparison correction. The black bars on the bottom right corner of microscope images represent 10 µm. (D) Summary of T2A::3xNLS::GFP vs. SL2::GFP::H2B comparisons across 6 different neuropeptide genes. Expression is determined using the reporter alleles in combination with NeuroPAL (Yemini et al., 2021), and the expression pattern of the SL2::GFP::H2B cassette more closely matches scRNA-seq data (Taylor et al., 2021).

## Description

In the course of analyzing the expression of neuropeptide-encoding genes (Sánchez et al., 2023), we found that different reporter cassettes engineered into endogenous loci, using the CRISPR/Cas9 system, yielded different outcomes. To analyze sites of reporter gene expression in the nervous system, characterized by a dense and often not entirely stereotyped localization of cells, it is beneficial to analyze spatially well-segregated nuclear reporter signals. The identity of nuclear-localized reporters can then, for example, be determined using the NeuroPAL reporter landmark strain (Yemini et al., 2021) . If the tagged protein is already nuclear, a straight fluorophore fusion achieves this goal. If the protein under investigation is, however, localized to specific subcellular sites outside the nucleus, or localized to the plasma membrane, or even secreted, it becomes necessary to (a) split the fluorophore from the protein under investigation and (b) target it to the nucleus via some nuclear localization signal.

In our efforts to analyze the expression of neuropeptide-encoding genes (Sánchez et al., 2023), we found that: (a) the two commonly used strategies for splitting off fluorophores – the “ribosomal skip” T2A peptide (Ahier and Jarriault, 2014) or the bicistronic SL2 linker (Tursun et al., 2009) – resulted in different intensities in fluorophore signals; (b) two different types of nuclear localization signals – 3x NLS (SV40) or addition of an H2B histone ( *his-44* ) - also yielded different signals in intensity. We did not consider the C-terminal NLS (EGL-13) in this study (Lyssenko et al., 2007) . (c) N- vs. C-terminal tagging also yields different signals in intensity in selected cases.

We arrived at this conclusion using two neuropeptide-encoding genes, *flp-20* and *nlp-51* . For both cases, we genome engineered four different sets of reporter cassettes ( **Fig.1A** ): (1) a T2A::3xNLS::GFP cassette inserted at the 3’end; (2) a T2A::GFP::H2B ( *his-44* ) cassette at the 3’end to assess differences in the localization signals; (3) a SL2::GFP::H2B cassette to assess T2A vs. SL2 and (4) an N-terminal GFP::H2B::SL2 cassette to compare N- vs. C-terminal tagging.

For both cases, we observed an allelic series. The most significant differences were observed between the two localization signals, with the GFP::H2B cassette being much brighter than the 3xNLS::GFP cassette ( Fig. 1B, C ). In some cases, such as for *nlp-51* expression in the AIM neuron, no signal was detected using the 3xNLS::GFP cassette while fluorescence was detected with the GFP::H2B cassette ( Fig. 1B ) and matched with scRNA-seq data (e.g., *nlp-51* in AIM) (Taylor et al., 2021) . In cases where fluorescence was detected in both cassettes, ~5-fold increases in fluorescence intensity were observed when using the GFP::H2B cassette ( Fig. 1B, C ). This could be the result of several factors, from more efficient nuclear transport (and hence greater nuclear concentration) by H2B to its greater stability to potential toxicity of multiplexed NLS(SV40) (Lyssenko et al., 2007) . Additionally, C-terminal SL2 yielded stronger signals than T2A ranging from a 34% to 2+ fold increase ( Fig. 1B, C ). This was surprising given previous work showing that genes downstream of the SL2 operon express at half of the level of the first gene (Cutter et al., 2009) , while proteins joined by T2A sequences are generally expressed stoichiometrically (Ahier and Jarriault, 2014) . Lastly, N-terminal tagging resulted in brighter fluorescence for *flp-20* and made no difference for *nlp-51* . It is unclear whether this is case-dependent or whether the fluorescence for *nlp-51* was reaching saturation and additional increases could not be detected.

Comparing T2A::3xNLS::GFP vs. SL2::GFP::H2B reporter tags for four additional genes ( *ins-6* , *ins-9* , *flp-32* , *flp-27* ) yielded similar results, with either higher fluorescence intensity or novel signals detected for the SL2::GFP::H2B cassettes ( Fig. 1D ). Overall, this suggests for endogenous CRISPR/Cas9 tagging to achieve maximum nuclear signal, SL2 linker and GFP::H2B are superior to other options and more closely align with scRNA-seq data from the CeNGEN project (Taylor et al., 2021) . In cases where both N- and C-terminal tagging are viable options, N-terminal tagging results in at least similar if not higher expression levels. Although SL2 linker and GFP::H2B resulted in brighter fluorophore expression, there may be specific scenarios where alternative options offer selective advantages. For example, visualization of fluorophore expression after T2A necessitates that the upstream gene (e.g., neuropeptide) is translated. Additionally, H2B overexpression can result in toxicity (Singh et al., 2010) , and its increased stability may not allow for the examination of fast temporal dynamics.

## Methods

All strains were raised at 20 ^o^ C, on nematode growth media (NGM) plates, and fed OP50 *Escherichia coli* as previously described (Brenner, 1974) . All neuropeptide GFP reporters were created by CRISPR/Cas9 genome engineering by SUNY Biotech. For full cassette sequences, please refer to Extended Data 1-4. For analysis of the reporter constructs, L1 animals were mounted on 5% agarose pads, immobilized with 100 mM sodium azide, and imaged on a Zeiss LSM880 using a 40X objective lens. GFP expression reporters were identified at single-neuron resolution as described (Yemini et al., 2021) . Fluorescence intensities were determined using the Zeiss Zen Blue 3.1 software, and plotted using GraphPad Prism 5. One-way ANOVA statistical tests were conducted followed by posthoc t-tests with Tukey correction.

## Reagents

**Table:** 

Strain	Worm Strain	Available from
PHX2805	*nlp-51(syb2805[nlp-51::T2A::3xNLS::GFP]) II*	CGC
PHX3983	*nlp-51(syb3983[nlp-51::T2A::GFP::H2B]) II*	CGC
PHX3936	*nlp-51(syb3936[nlp-51::SL2::GFP::H2B]) II*	CGC
PHX3997	*nlp-51(syb3997[GFP::H2B::SL2::nlp-51]) II*	CGC
PHX3241	*flp-20(syb3241[flp-20::T2A::3xNLS::GFP]) X*	CGC
PHX3995	*flp-20(syb3995[flp-20::T2A::GFP::H2B) X*	CGC
PHX4049	*flp-20(syb4049[flp-20::SL2::GFP::H2B]) X*	CGC
PHX4020	*flp-20(syb4020[GFP::H2B::SL2::flp-20::SL2]) X*	CGC
PHX2685	*ins-6(syb2685[ins-6::T2A::3xNLS::GFP]) II*	CGC
PHX5364	*ins-6(syb5463[ins-6::SL2::GFP::H2B]) II*	CGC
PHX2616	*ins-9(syb2616[ins-9::T2A::3xNLS::GFP]) X*	CGC
PHX5536	*ins-9(syb5536[ins-9::SL2::GFP::H2B]) X*	CGC
PHX3213	*flp-27(syb3213[flp-27::T2A::3xNLS::GFP]) II*	CGC
PHX4413	*flp-27(syb4413[flp-27::SL2::GFP::H2B]) II*	CGC
PHX3366	*flp-32(syb3366[flp-32::T2A::3xNLS::GFP]) X*	CGC
PHX4374	*flp-32(syb4374[flp-32::SL2::GFP::H2B]) X*	CGC

## Extended Data

Description: Sequence of T2A 3xNLS GFP cassette. Resource Type: Text. DOI: 10.22002/63mde-8ng94

Description: Sequence of T2A GFP H2B cassette. Resource Type: Text. DOI: 10.22002/mnx08-gs016

Description: Sequence of SL2 GFP H2B cassette. Resource Type: Text. DOI: 10.22002/xdrps-h1538

Description: Sequence of GFP H2B SL2 cassette. Resource Type: Text. DOI: 10.22002/q1n0z-y6s54
